# Analysing the health effects of simultaneous exposure to physical and chemical properties of airborne particles

**DOI:** 10.1016/j.envint.2015.02.010

**Published:** 2015-06

**Authors:** Monica Pirani, Nicky Best, Marta Blangiardo, Silvia Liverani, Richard W. Atkinson, Gary W. Fuller

**Affiliations:** a MRC-PHE Centre for Environment and Health, King's College London, Division of Analytical and Environmental Science, Franklin-Wilkins Building, 150 Stamford Street, SE1 9NH, London, UK; b MRC-PHE Centre for Environment and Health, Imperial College London, Department of Epidemiology and Biostatistics, 526 Norfolk Place, W2 1PG London, UK; c Brunel University, Department of Mathematics, UB8 3PH Uxbridge, London, UK; d MRC Biostatistics Unit, Institute of Public Health, Forvie site, Robinson Way, CB2 0SR Cambridge, UK; e Imperial College London, Department of Epidemiology and Biostatistics, 526 Norfolk Place, London W2 1PG London, UK; f MRC-PHE Centre for Environment and Health, St. George's University of London, Population Health Research Institute, Cranmer Terrace, SW17 0RE London, UK

**Keywords:** Airborne particles, Bayesian inference, Dirichlet process mixture model, Time series, Respiratory mortality

## Abstract

**Background:**

Airborne particles are a complex mix of organic and inorganic compounds, with a range of physical and chemical properties. Estimation of how simultaneous exposure to air particles affects the risk of adverse health response represents a challenge for scientific research and air quality management. In this paper, we present a Bayesian approach that can tackle this problem within the framework of time series analysis.

**Methods:**

We used Dirichlet process mixture models to cluster time points with similar multipollutant and response profiles, while adjusting for seasonal cycles, trends and temporal components. Inference was carried out via Markov Chain Monte Carlo methods. We illustrated our approach using daily data of a range of particle metrics and respiratory mortality for London (UK) 2002–2005. To better quantify the average health impact of these particles, we measured the same set of metrics in 2012, and we computed and compared the posterior predictive distributions of mortality under the exposure scenario in 2012 vs 2005.

**Results:**

The model resulted in a partition of the days into three clusters. We found a relative risk of 1.02 (95% credible intervals (CI): 1.00, 1.04) for respiratory mortality associated with days characterised by high posterior estimates of non-primary particles, especially nitrate and sulphate. We found a consistent reduction in the airborne particles in 2012 vs 2005 and the analysis of the posterior predictive distributions of respiratory mortality suggested an average annual decrease of − 3.5% (95% CI: − 0.12%, − 5.74%).

**Conclusions:**

We proposed an effective approach that enabled the better understanding of hidden structures in multipollutant health effects within time series analysis. It allowed the identification of exposure metrics associated with respiratory mortality and provided a tool to assess the changes in health effects from various policies to control the ambient particle matter mixtures.

## Highlights

• Health effects of PM mixtures were assessed using a Bayesian model.
• In London 2002-2005, days were clustered using particle and response profiles.
• An excess of respiratory mortality was associated with non-primary particles.
• A consistent reduction in the airborne particles was found in 2012 vs 2005.
• A decrease of respiratory mortality in 2012 was predicted vs 2005.

## 1 Introduction

Airborne particle matter (PM) is one of the air pollutants of primary health concern. Over the past two decades, PM mass metrics (e.g., particles with aerodynamic diameter < 10 μm, PM_10_, and particles with aerodynamic diameter < 2.5 μm, PM_2.5_) have received much attention, and many studies have shown that high concentrations of PM are associated with increased risks of mortality and morbidity. More recently, the evidence derived from studies of long- and short-term exposure has been judged sufficient to infer causality for fine particles (EPA, 2012; WHO/Europe, 2013).

The evidence for the association between PM and short-term adverse endpoints, derives largely from observational ecological time series studies (e.g., Bell et al., 2004; HEI, 2010; Bell et al., 2013; Atkinson et al., 2014; and references therein). Since the early 1990s the results from these studies have played an important role in setting standards for acceptable levels of ambient pollution. The quantification of the impact of air pollution on health has been historically undertaken through a single pollutant approach, using regression-based techniques, where the co-pollutants have been treated as modifying or confounding factors. This reliance on single pollutant results is due, in part, to measurement and source complexities (such as the intrinsic correlated nature of air pollutants) which have limited the development of statistically robust multipollutant models, and in part to the regulatory strategies of air quality management which have addressed a single pollutant at a time (Dominici et al., 2010).

Air pollution exists, however, as a heterogeneous mix of different compounds. In particular, airborne particulate is made up of a number of solid and liquid components, including acids (such as nitrates and sulphates), organic chemicals, metals, soil or dust particles, soot, allergens and smoke. These components also vary in number, size, shape, surface area, solubility and origin. Thus, estimation of how simultaneous exposure to multiple air pollutants affects the risk of adverse health response represents a challenge for scientific research and air quality management.

To gain better insight into the features of air pollution mixtures and their effect, there is a consequent need to explore new statistical methods able to integrate standard methodological tools for a better understanding of these complex systems. In a recent review of techniques for estimating health effects of multiple air pollutants, Oakes et al. (2014) highlighted that clustering of pollution profiles has been shown to be an effective approach.

Previous temporal clustering analyses have been successfully applied in air pollution exposure assessment, involving mainly heuristic methods such as agglomerative hierarchical clustering (Gu et al., 2012) and *k*-means partitioning clustering (Austin et al., 2012). Recently, *k*-means clustering solutions of air pollutants have also been used as covariates within health model effect estimation (Matyasovszky et al., 2011; Zanobetti et al., 2014).

Despite the increasing popularity of these methods, they have some well known drawbacks. First, they do not allow an assessment of the statistical properties of the solutions provided, for example they do not provide an assessment of clustering uncertainties. Moreover, because these methods are based on similarity/dissimilarity measures between objects that are essentially described in terms of distance (e.g., Euclidean distance), they require that the time series of each pollutant has exactly the same dimensionality (i.e., they do not allow the inclusion of records which have missing data). This can represent a limitation when applied to air pollution monitoring data.

Mixture models (McLachlan and Peel, 2000; McLachlan and Baek, 2010) have been proposed as an alternative to heuristic clustering techniques. Generally, model-based clustering methods are based on the idea that the data follow a finite mixture of probability distributions such that each component distribution represents a cluster. Frühwirth-Schnatter and Kaufmann (2008) showed that model-based clustering based on finite mixture models can be extended to time series in a quite natural way. In the air quality field, Gómez-Losada et al. (2014) applied a finite mixture model for characterising air pollution mixtures, using maximum likelihood, via the expectation-maximization algorithm.

A long-standing issue that finite mixture models share with many traditional clustering methods (e.g., *k*-means), is the a priori determination of the number of clusters. Different methods can be used to estimate the number of components (i.e., clusters), using for example model selection criteria. However, an alternative way to handle this problem is to adopt a Bayesian nonparametric modelling approach, where the number of mixture components is not fixed in advance, but is determined by the model and the data. These models can be implemented using a Dirichlet process (DP) (Ferguson, 1973; Antoniak, 1974), a stochastic process commonly used in Bayesian nonparametrics to model the uncertainty about the functional form of the distribution of the parameters in a model. The support of the DP is restricted to discrete distributions and this results in a clustering effect that avoids the selection of a pre-defined number of clusters.

In this paper we propose an approach within the Bayesian paradigm to analyse the impact of multiple particle metrics on daily mortality, using the DP mixture model. Specifically, we provide a model that addresses, in a one-step procedure, both dimensionality reduction and regression. Our approach builds on the work of Molitor et al. (2010, 2011) which represents an alternative inferential approach to regression models when the covariates in analysis are correlated. The model, known as profile regression, performs a Bayesian clustering of the covariates by identifying exposure profiles and, simultaneously, links these to a response variable in non-parametric form (even though the model continues to be parametric within clusters). Profile regression has found further applications in epidemiology and in genomics (Papathomas et al., 2011, 2012; Hastie et al., 2013). In this paper we extend this technique to analyse time series data, accounting for their typical features like trends, seasonality and temporal components through smooth functions. The resulting probabilistic solution groups time points with similar multipollutant and response profiles.

To demonstrate our approach, we used daily particle metric data from London (UK) 2002–2005 and daily number of deaths from respiratory diseases (Atkinson et al., 2010). Additionally, to asses the recent efforts in reducing air pollution in London, we also predicted a mean response profile for mortality in the year 2012. Using measurements collected at the same monitoring site, we compared the predictive distribution of mortality in 2012 against the one computed in 2005.

## 2 Material and methods

### 2.1 Description of the data

Atkinson et al. (2010) described results from an epidemiological time series study examining the effect of different metrics of particulate collected in London, on cardiorespiratory hospital admission and mortality using univariate log-linear Poisson models. We selected a subset of exposure data for the period January 2002 to December 2005 (years 2000–2001 were excluded due to pour data availability; for anions the proportion of missing data was about 96%), and respiratory-related mortality as the outcome. To predict respiratory mortality given the multipollutant scenario that London experienced in 2012, we measured the same set of particle metrics that were recorded in 2002–2005.

#### 2.1.1 Mortality data

Daily count of deaths from respiratory diseases of London residents (2002–2005) were obtained from the Office for National Statistics and coded using the International Classification of Diseases, 10th Revision (ICD-10: Chapter J).

#### 2.1.2 PM measurements 2002–2005

Daily average concentrations of particle metrics included: particle number concentration (PNC), inorganic anions such as chloride, nitrate and sulphate, black smoke (BS) and gravimetric measurements of PM, such as PM_10_, PM_2.5_ and PM coarse fraction (that is, PM_10–2.5_ obtained by subtraction). With the exception of BS, the daily concentrations were obtained from a single background monitoring station in central London (North Kensington). BS was an average across several urban and suburban stations. PNC was measured using a TSI 3022A condensation particle counter, where particles are enlarged by condensation of saturated butanol vapour which are then counted using a laser and optical detector. The PM_10_ 24-hour filter samples were collected at 16.7 l per minute on quartz fibre filters using Partisol 2025 (Thermo) instruments and these filters were analysed by ion chromatography. Finally, daily average gravimetric PM_10_ and PM_2.5_ were sampled using a Partisol sampler and measured using methods in EN12341 and EN14907.

The data set also included PM apportioned into primary and non-primary sources (Fuller et al., 2002; Fuller and Green, 2006), giving modelled primary PM_10_ (PPM_10_), and non-primary PM subdivided by size fraction: non-primary PM_10_ (NPPM_10_), non-primary PM_2.5_ (NPPM_2.5_), and non-primary PM coarse fraction (NPcoarse). The source apportionment model assumed that primary PM_10_ was associated with nitrogen oxide (NO_x_) sources and the non-primary component was the fraction of PM not associated with NO_x_. NO_x_ is generally considered a robust marker for traffic pollution (Krzyzanowski et al., 2005).

#### 2.1.3 PM measurements 2012

For the year 2012, the PM measurements (except BS) were collected at the same background monitoring station in central London. Between 2005 and 2012, gravimetric filter substrates were changed from quartz fibre to PTFE coated glass fibre (Emfab, Pall). Because BS is no longer measured in London, we computed daily mean of BS from equivalent measured black carbon by aethalometer (Magee Scientific) at two background monitoring sites, and applied an adjustment factor of 0.27 following Heal and Quincey (2012).

#### 2.1.4 Confounding factors

Ecological time series studies are subject to complex forms of confounding (e.g., Peng et al., 2006; Bhaskaran et al., 2013). Typically, time series studies of mortality and morbidity control for long-term trends, seasonality, and time-varying factors, including climatology, which can potentially confound the association between an adverse health effect and polluted air. In our model, calendar time and temperature were considered as confounding variables and assumed to potentially influence the response variable via smooth functions.

Specifically, for all of the smooth functions we used natural cubic spline bases, in which the degree of smoothness was determined by the degrees of freedom (*df*). The choice of the *df* was based on the examination of the partial autocorrelation function of residuals and by minimization of the Akaike's Information Criterion (Akaike, 1973) and the Bayesian Information Criterion (Schwarz, 1978), fitting a log-linear Poisson regression model. We specified 32 *df* (8 *df* per year) for the smooth function of time and 3 *df* for the smooth function of temperature. In a previous study performed to investigate the potential for bias in estimating the short-term effects of air pollution, Shaddick et al. (2013) used London's 2002–2005 respiratory mortality and PM_10_ concentrations and showed that a similar adjustments provided an adequate balance between ensuring control for temporal trends and seasonal cycles as well as temperature, while leaving sufficient information for estimating the exposure effects.

For our study, hourly temperatures were downloaded from the London Air Quality Network using the R library *openair* (version 0.9-2) (Carslaw and Ropkins, 2012) and averaged on daily temporal scale. During the years 2002–2005, daily average temperature ranged from − 0.88 °C to 28.87 °C. We generated the B-spline basis matrices for calendar time and temperature outside the model, using the function *ns* of the R library *spline*, and we entered them as data.

#### 2.1.5 Data preparation

The exposure data were normalised to be on a comparable scale adopting the modified z-score recently proposed by Austin et al. (2012). Let *x*_*t*,*p*_ be the measurement on day *t* of particle metric *p*, for *t* = 1,…,*T* and *p* = 1,…,*P*. We transformed the original measurements as *z*_*t*,*p*_ = (*x*_*t*,*p*_ − Median(*x*_*p*_))/(Median(|*x*_*t*,*p*_ − Median(*x*_*p*_)|)).

In the previous analysis, Atkinson et al. (2010) observed associations for respiratory mortality with 1–day lag secondary PM masses. The estimated regression coefficients were obtained fitting separate univariate log-linear Poisson models. To study the value added by our new approach, we considered the previous study of Atkinson et al. (2010) as benchmark and thus the 1 day lag was chosen as the exposure window for particles.

### 2.2 Profile regression model for time series of multiple particles and health events

The proposed model is based on the DP, a popular tool for Bayesian nonparametric analysis, which relies on mixtures to represent distributions in the data. In Section S1 of the Supplementary material we provide a brief review of the DP mixture model.

Denote by *t* = 1,…,*T* a series of temporal points. Let the data consist of realizations of a response data vector *y* = (*y*_1_,…,y*_T_*), a set of (normalised) covariates (i.e., predictors) *z*_*t*,*p*_, *p* = 1,…,*P*, and a collection of confounding factors *u*_*t*,*h*_, *h* = 1,…,*H*. In our study, *y_t_* denotes the count number of deaths for respiratory diseases on day *t*, *z*_*t*_ = (*z*_*t*,1_,…,*z*_*t*,*P*_)′ represents a daily covariate profile of air particles, and *u*_*t*_ = (*u*_*t*,1_,…,*u*_*t*,*H*_)′ is a B-spline basis matrix for natural cubic splines of calendar time and temperature.

We assumed a joint probability model for the data, which takes the following form:

$$p\left(y_{t},z_{t}|\Theta ,u_{t}\right)=\sum_{k=1}^{\infty }w_{k}p\left(y_{t}|\Theta_{k},\Theta_{0},u_{t}\right)p\left(z_{t}|\Theta_{k},\Theta_{0}\right)$$

where *w_k_* are the mixture probabilities satisfying ∑_*k* = 1_^∞^*w*_*k*_ = 1 almost surely and indicating the probability of belonging to the *k*th component. *Θ* denotes the collection of model parameters, that includes component specific parameters *Θ*_*k*_ and global parameters *Θ*_0_, that is, *Θ* = (*Θ*_*k*_,*Θ*_0_).

The inference for such mixture models can be simplified by introducing latent variables that indicate the group memberships of objects (i.e., the cluster to which day *t* belongs to). We define these latent group labels as: *g* = (*g*_1_,…,*g*_*T*_), such that *p*(*g*_*t*_ = *k*) = *w*_*k*_. Thus, *g_t_* is chosen using a multinomial distribution parameterised by the mixing probabilities, *g*_*t*_|*w* ~ Multinomial(*w*).

Rather than specifying a parametric distribution for the mixture probabilities, *w_k_*, we modelled them as unknown quantities to be estimated by the data. Specifically, we assumed that *w_k_* are generated using a stick-breaking representation of the DP given by Sethuraman (1994). The name of this construction derives from an analogy given by breaking pieces off from a stick of unit length, where the breakpoints are randomly sampled from the Beta distribution. The mixture probabilities break the stick into a potentially infinite number of pieces, such that ∑_*k* = 1_^∞^*w*_*k*_ = 1. The first mixture probability is equal to *V*_1_, i.e., *w*_1_ = *V*_1_, where *V*_1_ ~ Beta(1,*α*) and for *k* ≥ 2 the *k* ‐th mixture probabilities are given by *V*_*k*_∏_*i* = 1_^*k* − 1^(1 − *V*_*i*_). We used a Gamma distribution to specify prior uncertainty for the precision parameter of DP (following Escobar and West (1995)), namely *α* ~ Gamma(*a*,*b*), where *a* = 2 and *b* = 1 are the shape and the inverse-scale (rate) parameter respectively.

We assumed a multivariate normal distribution for the *P* covariates:

$$p\left(z_{t}|\Theta_{k},\Theta_{0}\right)={\left(2\pi \right)}^{-\frac{P}{2}}|\Sigma_{k}|{}^{-\frac{1}{2}}\exp\left\{-\frac{1}{2}{\left(z_{t}-m_{k}\right)}^{\prime }\Sigma_{k}^{-1}\left(z_{t}-m_{k}\right)\right\}$$

where *m*_*k*_ = (*m*_*k*,1_,…,*m*_*k*,*P*_) is the mean vector for component *k* (i.e., location parameters), and *Σ*_*k*_ is the *P* × *P* symmetric positive-definite variance–covariance matrix.

We specified hyperpriors for *m_k_* and *Σ*_*k*_ similar to Molitor et al. (2011), adopting an empirical Bayesian approach. We assumed a normal distribution for the location parameters, that is, *m*_*k*_ ~ N(*m*_0_,*Σ*_0_) (with *m*_0_ equal to the empirical mean of each covariate, and *Σ*_0_ having a diagonal structure with elements equal to the square of empirical range of each covariate). We specified a Wishart distribution for the precision matrix *Q*_*k*_ = *Σ*_*k*_^− 1^ (i.e., inverse variance–covariance matrix), that is, *Q*_*k*_ ~ *W*(*Φ*,*ν*), where *Φ* is a symmetric (non-singular) matrix parameter (set equal to the inverse of the empirical variance multiplied by 1/*P*) and *v* is the degrees of freedom parameter (set equal to *P*).

The response was modelled as a Poisson:

$$p\left(y_{t}|\Theta_{k},\Theta_{0},u_{t}\right)=\frac{\lambda_{t}^{y_{t}}}{y_{t}!}\exp\left(-\lambda_{t}\right)$$

where

$$\lambda_{t}=E_{t}\exp\left(\mu_{t}\right)$$

and

$$\mu_{t}=\mu_{k}+\sum_{h=1}^{H}f_{h}\left(u_{t,h},df_{h}\right)+\varepsilon_{t}$$

assuming *ε_t_* be normal distributed with zero mean and variance *σ*_*ε*_^2^. Here *μ_t_* is the mean response for day *t* and *E_t_* is the expected offset given by the average number of deaths for respiratory diseases in the full period in study.

The parameter of interest is *μ_k_*, which represents the log relative risk for the outcome of interest associated with the *k*th cluster, were each cluster includes days with similar multipollutant profile. The functions *f*(⋅,*df*_*h*_) denote smooth functions of confounding factors, with smoothing parameters *df_h_*. The basis functions are associated with the relative coefficients *β*_1_,…,*β_H_*, that we assumed follow a weakly informative Student-*t* prior distribution, with location, scale and degree of freedom set to 0, 2.5 and 7 respectively (Gelman et al., 2008), that is *β* ~ *t*_7_(0,2.5). The smooth functions were constrained to only have a global effect on the response and not a cluster-specific effect.

#### 2.2.1 Predictions

Classical regression provides concentration response functions that can be used in health impact assessment or to assess the costs and benefits of policies to decrease pollution exposures. By using profile regression we could identify and quantify the types of daily pollutant mixtures that are associated with adverse health effects. We could also analyse what would happen to this health outcome if the exposure variables were changed. This was accomplished by a predictive approach (Müller et al., 1996). The main idea here was to obtain a posterior predictive distribution of the response, given a new exposure scenario. In our application the simulated predictions represented an average effect of the changed air particle mixtures in London.

We compared two predictive scenarios based on: (i) concentrations of particles measured in 2005, and (ii) concentration of the same particles measured in 2012, to analyse any changes in respiratory mortality arising from the combined effects of local, city, national and EU policies to manage air pollution in interval of seven years period.

The posteriors predictions were carried out using the method proposed by Liverani et al. (forthcoming) We refer the reader to Section S2 of the Supplementary Material for the description of the prediction computation.

Before computing predictions of mortality for the different exposure scenarios, we used a predictive cross-validation technique as model checking. We partitioned the four years time series, using the data collected in 2002–2004 as training sample and the data in 2005 as validation sample. We predicted the respiratory deaths for the 2005 and we compared the validation predictions with the actual observations.

We then used the full time series data and computed the posterior predictive distribution of the count of respiratory-related deaths in 2012, and we compared this with the one computed for the year 2005. Finally, we quantified an average reduction in mortality attributable to the decrement of the ambient air particles analysing the distribution of the percent change between the two years.

#### 2.2.2 Posterior computation, convergence and sensitivity analysis

Inference for the model relies on MCMC computational methods. We used a slice dependent sampler algorithm for posterior computation, as implemented in the R package *PReMiuM* (version 3.0.24) (Liverani et al., forthcoming). Slice sampling methods go back to Neal (2003), and are successively described for DP mixture models by Walker (2007), Kalli et al. (2011). The basic idea is to introduce an auxiliary latent slice variable that allows a finite number of clusters to be sampled within each iteration of the sampler. The algorithm implemented in *PReMiuM* combines a Gibbs sampler with Metropolis-within-Gibbs steps. It also implements label switching moves as suggested by Papaspiliopoulos and Roberts (2008).

The algorithm was run for 70,000 iterations with the first 20,000 discarded as burn-in. Using 1 in 10 thinning, this gave us a total of 5000 draws from the posterior distribution of parameters and predictions.

Convergence was checked through the inspection of trace plots of the samples, the estimated kernel density plots and the autocorrelation plots of the main global parameters of the model using the R package *coda* (version 0.16-1).

We also checked the robustness of the results under different initializations (i.e., different initial number of clusters).

Finally, we performed a sensitivity analysis with respect to changes in the prior for the DP precision parameter, *α*, a hyperparameter that influences the number of clusters (i.e., mixture components). We considered two different Gamma prior distributions for *α*, with *α* = 2, *b* = 4 and *a* = 1, *b* = 1.

#### 2.2.3 Post-processing

To summarise the features of the rich output from the MCMC sampler, we performed a post-processing of the posteriors, as suggested by Molitor et al. (2010, 2011), that relied on a *representative* partition (i.e., that is most supported by the data) obtained by using a similarity matrix based upon the output of the MCMC. At each iteration of the sampler, we recorded a *T* × *T* score matrix with (*i*,*j*)th elements set equal to 1 if day *i* and day *j* belong to the same cluster and 0 otherwise. The end of this process leads to a probability matrix, *S*, formed by averaging the score matrices obtained at each iteration, thus element *S*_*i*,*j*_ denotes the probability that day *i* and *j* are assigned to the same cluster. We used a clustering procedure partitioning around medoids (Kaufman and Rousseeuw, 2005) on the dissimilarity matrix 1 − *S* to obtain representative partitions. Once the representative clustering was defined, a model averaging approach was adopted to evaluate the uncertainty related to the characteristics of the clusters that involved running through the MCMC run, obtaining an average value for the model parameters (effects and cluster related parameters) across all days in a certain cluster.

## 3 Results

Summary statistics for deaths for respiratory-related diseases and ambient air particles measured in London in the years 2002–2005 are given in Table 1. Section S3 of the Supplementary material shows also the time series of daily respiratory mortality and daily mean particle concentrations.

The correlations between the daily concentrations of pollutants showed different degrees of interdependence in these metrics, as shown in Table 2.

**Fig. 1:**
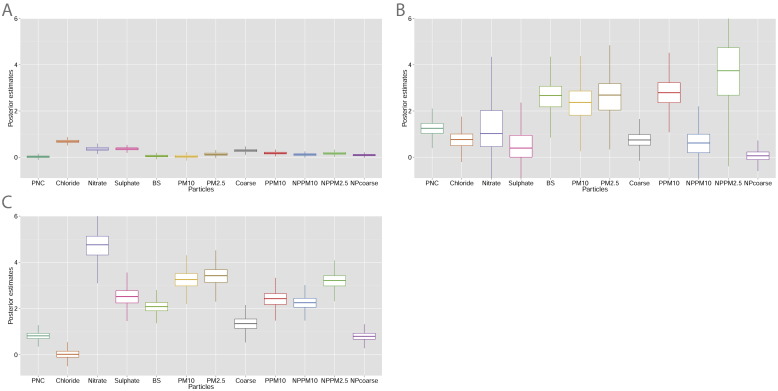
Box plots showing the distribution of the posterior means for each particle component (on normalised scale) for the three clusters that form the representative clustering (A = cluster 1; B = cluster 2; C = cluster 3).

The representative clustering separated the days into three main clusters, which included respectively 1156, 63 and 242 days. Fig. 1 shows the posterior distributions for the particle metrics (on normalised scale) by cluster, while Table 3 displays a summary of the cluster multipollutant profiles on their original scale.

Compared to clusters 1 and 3, cluster 2 had larger posterior errors as the number of days included was lower.

The risk of mortality for respiratory diseases varied according to these cluster profiles.

Cluster 1 was characterised by low posterior estimates for most of the particles (except chloride), and had the lowest risk of mortality when compared to the average mortality in 2002–2005. The posterior relative risk of mortality, *μ*_1_, associated with this cluster was 0.98 (95% credible intervals (CI): 0.96,1.00).

Cluster 2 was characterised by low posterior estimates of inorganic anions and secondary particles and higher posteriors for primary emissions, with a posterior relative risk of mortality, *μ*_2_, equal to 1.00 (95% CI: 0.97, 1.03). This cluster included mainly winter days.

Finally, cluster 3 was dominated by secondary aerosol, especially nitrate and sulphate, with high posteriors of non-primary airborne particles. We found a posterior relative risk of mortality, *μ*_3_, equal to 1.02 (95% CI: 1.00, 1.04). This third cluster included mainly spring and autumn days.

**Fig. 2:**
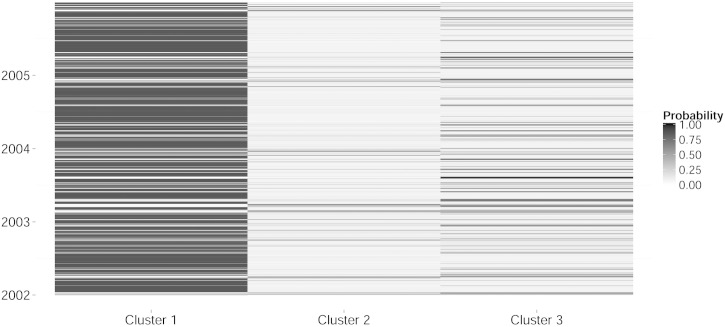
Heatmap of posterior probability that day *t* belongs to one of the three representative clusters.

Fig. 2 displays the heatmap of the posterior probabilities that the days (period: 2002–2005) were included in a cluster. For this data set, we found that the days exhibited a high probability of being assigned to a specific cluster.

**Fig. 3:**
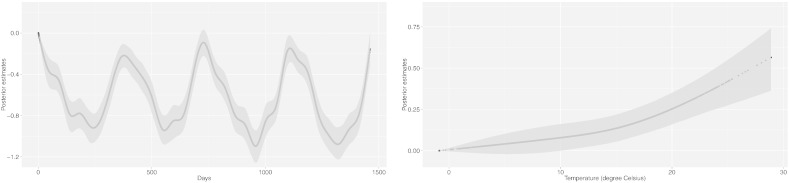
Posterior estimates (mean and 95% CI) for the coefficients of the natural cubic spline of time (left panel) and natural cubic spline of temperature (right panel).

We also analysed the posterior estimates for the coefficients associated with the design matrices of B-splines of time and temperature for controlling for seasonal and long-term trend and weather conditions. The posterior mean and the 95% CI of the estimated coefficients are displayed in Fig. 3, showing the effective capability of the model to depict the non-linear effect of these factors.

### 3.1 Prediction results

Firstly, we performed a cross-validation analysis to check the fit of our model, as reported in Section S4 of the Supplementary material. Secondly, we examined the combined effect of these particles computing the predictive distribution of the respiratory mortality counts under the exposure scenario given by the concentrations of particles measured in 2012. Then we compared this with the predictive distribution obtained for 2005.

Table 4 describes the summary statistics for the airborne particles measured in 2012. A large reduction in airborne particles from 2002–2005 to 2012 is clearly visible. This arose mainly from decreases in regional non-primary PM (mainly secondary sulphate and nitrate) rather than London specific policies that would have had greater impact on primary PM and BS, consistent with the earlier findings of Fuller and Green (2006). The large decrease in PNC was most likely due to a decrease in the sulphur content of diesel in 2008 which also contributed to decreased sulphate concentrations (Jones et al., 2012).

Comparing the predictive distribution of the deaths for 2012 vs 2005, we found a reduction in respiratory mortality, corresponding to an average percentage change in the posterior predictive distributions of − 3.51% (95% CI: − 0.12%, − 5.74%). Based on the observed number of deaths for respiratory-related diseases which occurred in 2005, we would expect an average reduction in mortality of approximately 270 subjects.

### 3.2 Sensitivity analysis results

The different priors on the precision parameter *a* turned out not to have relevant impact on the clustering result. The prior specification of Gamma distribution with: (i) *a* = 2 and *b* = 4 produced a median of 14 clusters, however only three clusters per sweep were well populated (the others included ≤ 9 days); (ii) *a* = 1 and *b* = 1 produced a median of 11 clusters, but again only three clusters per sweep were well populated. The results essentially confirmed the reliability of the three representative clusters obtained in the post-processing.

The diagnosis performed setting different starting points in the number of clusters in the initialization of the model, showed the consistency of the results.

## 4 Discussion

There is an increasing need to assess the health effects of multiple air pollution exposures for both health research and air quality management. This requires new statistical methods to better understand these complex systems.

We addressed this problem by introducing a Bayesian modelling framework that offers a flexible way to model the joint distribution of a response and pollutants. The proposed model is based on the DP mixture models that represent an appealing tool for clustering data. In standard applications, however, these models assume that the observations are exchangeable and the data points do not have an inherent order influencing their labelling. Several Bayesian nonparametric studies have been specially targeted to clustering temporally evolving phenomena. For example, in a recent work Nieto-Barajas and Contreras-Cristán (2014) accommodated the temporal effects in time series data using a first order autoregressive process. In our model, we used a simple and feasible solution given by introducing natural cubic splines that correct for temporally dependent confounding effects, adjusting for seasonal and long-term trends and weather variables such as temperature.

A clear benefit of our model is the simultaneous estimation of the contribution of all pollutants to the mortality risk. This would allow policy makers to have a holistic picture of the effect of complex air pollution mixtures. This is a novel feature of our model, in comparison to the recent two-stage approaches proposed by Matyasovszky et al. (2011) and Zanobetti et al. (2014) for example. Our model, moreover, presents additional advantages compared with traditional clustering methods. First, it is able to address the challenging question of uncertainty in the cluster assignment. In our application we found that the uncertainty associated with the partitioning of the days to clusters was quite low, and this supports the use of the partitioning around medoids method on the posterior dissimilarity matrix to obtain a representative partition. Once this partition was obtained, full uncertainty about its characteristics was recovered from post processing of the full MCMC output. Second, because of the Bayesian computation method adopted, we could consider the whole time series of particles, without the exclusion of days with missing measurements. In fact, missing values in a (daily) covariate profile were sampled within the MCMC sampler (i.e., it checks which cluster the day is allocated to and then samples). Finally, our model was able to uncover clusters in the data naturally, without a clustering structure being imposed by the user.

However, compared to non-Bayesian methods, our model had higher computational cost. Specifically, for the inferential procedures, it took approximately 25 min using an Intel(R) Core i7 CPU machine (2.40 GHz, 8 GB RAM). We think that this sacrifice in terms of computational effort is reasonable, given the advantages provided by our Bayesian approach with MCMC inference.

We applied our model to a real data set, in which we studied the temporal structure of particle mix in London and its effect on respiratory mortality. It identified which type of pollutant mixtures were associated with mortality and quantified the risk; in this case a relative risk of mortality was 1.02 (95% CI: 1.00, 1.04) on days with increased secondary PM mass concentrations including high concentrations of inorganic PM such as sulphate and nitrate.

The association of adverse health effects with secondary PM masses (i.e., metrics not associated with NO*_x_* sources) was consistent with Atkinson et al. (2010).

Particulate nitrate and sulphate are acidic in nature. Nitrate is mainly the product of oxidation of nitrogen oxides (which sources include fossil-fuel combustion; road transport, space heating and aircraft for example, biomass burning, soil release and ammonia oxidation from agriculture), while sulphate is mainly from the oxidation of sulphur dioxide (emitted from power plants and industrial facilities and to a lesser extent natural sources such as oceans, plant and soils, and volcanoes along with ammonia oxidation). Evidence of associations between secondary inorganic PM, such as sulphates and nitrates with negative health effects are limited and still insufficient to support a causality (Reiss et al., 2007; WHO/Europe, 2013). However, the results of our study for respiratory mortality are consistent with (Ostro et al., 2009), which observed an increased risk of respiratory hospital admissions in children associated with an increase in sulphate for a 3-day lag. Recently, Dai et al. (2014) found that particle sulphur modified the effect of PM_2.5_ on total and respiratory mortality. As sulphate is the primary form of particle sulphur, the authors interestingly argued about the plausibility of the health effects of sulphate, supported by toxicology findings that show, for example, that it is linked to an increased oxidative stress and coagulation (Chuang et al., 2007). Cao et al. (2012) found significant positive associations of total, cardiovascular, and respiratory mortality with different PM components, including nitrate, at 1 day lag.

Rather than producing single-pollutant concentration response functions for use in health impact assessment or to assess the cost benefits of policies to decrease pollution exposures, our approach provides a predictive tool to allow the assessment of changes in the pollutant mixture. This is a far more realistic representation of the outcomes of the range of policies being employed across different emissions sectors at different spatial and government levels rather than taking a single pollutant approach. When assessing impact through a single pollutant approach, it is unclear if the concentration response function for a single pollutant is acting as a tracer for health effects from other correlated pollutants; for instance Janssen et al. (2012) have examined if black carbon particles or PM mass concentrations are a better metric for airborne particle health effects. These issues are avoided by instead looking at mixtures. As an illustration of this approach we estimated the changes in health response from changes in pollution concentrations in all 12 exposure variables measured in our data set. Between 2005 and 2012 we predicted a decrease in annual respiratory mortality of − 3.51% (95% CI: − 0.12%, − 5.74%) in London.

Our study has several limitations. It was ecological and the measurements of particle metrics were collected at a single monitoring site in central London, therefore we could not account for individual features and activities. It is commonly accepted that in population-based time series studies, individual risk factors (age, diet, smoking etc.) are unlikely to be confounders as they do not vary temporally with air pollution over relatively short-term periods (e.g., Burnett et al., 2003). However, the ambient measurements used in our study could lack of spatial and temporal resolution due to individual's activities (Özkaynak et al., 2013) and generally be less representative than personal monitoring for assessing particulate exposure (Buonanno et al., 2013). Moreover, respiratory mortality in London population was related only to outdoor particle concentrations, while people spend considerable time in indoor environments and exposure highly depends on indoor concentrations (Morawska et al., 2013).

At the time of the original study of Atkinson et al. (2010), only limited information on PM composition were available. A more in-depth understanding of the dynamics in pollution mixtures will be provided by the future inclusion of organic and elemental carbon along with metal species and oxidative potential. Finally, this study has only considered daily mortality from respiratory causes but it could equally be applied to other outcomes, namely daily cardiovascular mortality and cardiorespiratory hospital admissions.

## 5 Conclusion

The proposed modelling approach overcomes many of the challenges in estimating the adverse health effects of mix of polluted air. It allowed the inclusion of different correlated exposure metrics and, in comparison to traditional clustering methods, it presented a number of attractive advantages. Among the others, it incorporated the association with the health outcome in determining the pollutant profile that characterises cluster membership.

This approach could provide new technique for policy makers to assess the impact of interventions that affect the mixture rather than individual pollutants. This reflects the reality of air pollution management strategies. For instance, the progressive restrictions on vehicle emission through euro-standards have acted on several pollutant simultaneously.

In our application, we found that cluster membership seemed to be an effect modifier in health effects analysis, denoting pollutant mixtures that could be targeted as part of air quality control strategy for health.

## Conflict of interest statement

The authors have no conflicts of interest to disclose.

## Figures and Tables

**Table 1:** Descriptive statistics of respiratory mortality and airborne particle metrics. London, 2002–2005.

			Percentiles		
Variables	Mean	Range	25th	50th	75th
Deaths (per day)	21.60	6.00–58.00	16.00	21.00	26.00
PNC (cm^− 3^/1000)	21.19	5.39–52.44	14.63	19.97	25.91
PM component					
Chloride (μg/m^3^)	1.31	0.01–9.06	0.25	0.88	1.98
Nitrate (μg/m^3^)	3.77	0.03–30.89	1.35	2.44	4.47
Sulphate (μg/m^3^)	2.93	0.23–20.63	1.51	2.25	3.89
BS (μg/m^3^)	6.23	1.40–31.33	4.00	5.40	7.60
PM size					
PM_10_ (μg/m^3^)	26.63	5.00–119.00	17.00	23.00	32.00
PM_2.5_ (μg/m^3^)	18.85	1.00–104.00	11.00	15.00	22.00
Coarse (μg/m^3^)	7.89	0–33.00	5.00	7.00	10.00
PM source apportionment					
PPM_10_ (μg/m^3^)	4.63	0.80–39.10	2.50	3.70	5.60
NPPM_10_ (μg/m^3^)	11.50	0–61.00	7.00	9.90	14.20
NPPM_2.5_ (μg/m^3^)	5.75	0–32.60	2.40	4.20	7.40
NPcoarse (μg/m^3^)	5.99	0–42.20	4.00	5.60	7.40

**Table 2:** Correlation between pairs of airborne particle metrics. London, 2002–2005.

	PNC	Chloride	Nitrate	Sulphate	BS	PM_10_	PM_2.5_	Coarse	PPM_10_	NPPM_10_	NPPM_2.5_	NPcoarse
PNC												
Chloride	0.34											
Nitrate	0.38	− 0.17										
Sulphate	0.08	− 0.31	0.52									
BS	0.49	− 0.16	0.46	0.35								
PM_10_	0.30	− 0.16	0.67	0.66	0.48							
PM_2.5_	0.31	− 0.29	0.70	0.68	0.51	0.91						
Coarse	0.09	0.11	0.18	0.25	0.13	0.57	0.26					
PPM_10_	0.72	− 0.09	0.53	0.30	0.74	0.53	0.56	0.15				
NPPM_10_	− 0.12	− 0.16	0.43	0.55	0.20	0.68	0.60	0.49	0.11			
NPPM_2.5_	− 0.16	− 0.39	0.48	0.68	0.28	0.67	0.68	0.31	0.14	0.86		
NPcoarse	0.02	0.22	0.21	0.15	0.03	0.43	0.25	0.56	0.06	0.71	0.31	

**Table 3:** Summary of cluster profiles (on original scale): distribution means (95% CI) for characteristics of clusters from the representative clustering.

Particle compounds	Cluster 1 (1156 days)	Cluster 2 (63 days)	Cluster 3 (242 days)
PNC (cm^− 3^/1000)	20.08 (19.54, 20.67)	27.01 (23.63, 30.42)	24.56 (22.58, 26.51)
Chloride (μg/m^3^)	1.38 (1.28, 1.47)	1.43 (0.95, 1.90)	0.90 (0.62, 1.21)
Nitrate (μg/m^3^)	2.90 (2.73, 3.41)	3.76 (2.19, 7.74)	8.58 (6.49, 9.90)
Sulphate (μg/m^3^)	2.61 (2.49, 2.79)	2.65 (1.73, 4.54)	4.76 (3.94, 5.50)
BS (μg/m^3^)	5.48 (5.33, 5.76)	9.80 (7.59, 11.57)	8.83 (7.65, 9.82)
PM_10_ (μg/m^3^)	23.16 (22.51, 25.48)	37.24 (26.94, 45.09)	42.52 (37.61, 47.25)
PM_2.5_ (μg/m^3^)	15.65 (15.12, 17.40)	28.45 (19.10, 35.12)	32.09 (26.84, 35.82)
Coarse (μg/m^3^)	7.57 (7.32, 7.88)	8.87 (7.23, 10.57)	10.36 (8.82, 12.00)
PPM_10_ (μg/m^3^)	3.95 (3.82, 4.22)	7.61 (5.95, 9.70)	7.10 (5.79, 8.06)
NPPM_10_ (μg/m^3^)	10.27 (9.97, 10.73)	11.93 (7.68, 15.86)	17.32 (15.21, 19.46)
NPPM_2.5_ (μg/m^3^)	4.56 (4.34, 5.01)	12.04 (5.41, 18.76)	10.90 (8.74, 12.27)
NPcoarse (μg/m^3^)	5.76 (5.61, 5.91)	5.70 (4.87, 6.63)	6.96 (6.12, 7.86)

**Table 4:** Descriptive statistics of airborne particle metrics. London, 2012.

			Percentiles		
Variables	Mean	Range	25th	50th	75th
PNC (cm^− 3^/1000)	12.12	5.34–25.02	9.16	11.49	14.57
PM component					
Chloride (μg/m^3^)	1.37	0.20–6.40	0.50	1.10	1.80
Nitrate (μg/m^3^)	3.33	0.10–34.40	0.70	1.60	4.00
Sulphate (μg/m^3^)	1.67	0.20–13.50	0.80	1.30	2.10
BS (μg/m^3^)	5.88	1.11–27.78	3.33	4.44	7.41
PM size					
PM_10_ (μg/m^3^)	17.70	4.00–76.00	11.00	14.00	20.75
PM_2.5_ (μg/m^3^)	11.31	2.00–61.00	6.00	8.00	13.00
Coarse (μg/m^3^)	6.60	0–31.00	4.00	6.00	8.00
PM source apportionment					
PPM_10_ (μg/m^3^)	4.11	1.00–14.40	2.30	3.20	5.30
NPPM_10_ (μg/m^3^)	9.49	1.17–29.61	6.12	8.46	11.88
NPPM_2.5_ (μg/m^3^)	3.42	0–17.54	1.35	2.63	4.33
NPcoarse (μg/m^3^)	6.40	0.24–13.47	4.69	6.21	8.00

## Appendix A Supplementary data

Statistical framework, predictions, time series plot and cross-validation.
