# Supplementary material for: Analysing the health effects of simultaneous exposure to physical and chemical properties of airborne particles
Source: Environ Int. 2015 Jun;79:56–64. doi: 10.1016/j.envint.2015.02.010 (PMC4396698; doi:10.1016/j.envint.2015.02.010)
Supplement: Supplementary file 1 — Statistical framework, predictions, time series plot and cross-validation. [file mmc1.pdf]

Supplementary material for: Analysing the health effects of simultaneous exposure to physical and chemical properties of airborne particles

Monica Pirani[a,*], Nicky Best[b], Marta Blangiardo[b], Silvia Liverani[c,d,e], Richard W. Atkinson[f], Gary W. Fuller[a]

[a] *MRC-PHE Centre for Environment and Health, King's College London, Division of Analytical and Environmental Science, Franklin-Wilkins Building, 150 Stamford Street, SE1 9NH, London, UK*
[b] *MRC-PHE Centre for Environment and Health, Imperial College London, Department of Epidemiology and Biostatistics, 526 Norfolk Place, W2 1PG, London, UK*
[c] *Brunel University, Department of Mathematics, UB8 3PH, Uxbridge, London, UK*
[d] *MRC Biostatistics Unit, Institute of Public Health, Forvie site, Robinson Way, CB2 0SR, Cambridge, UK*
[e] *Imperial College London, Department of Epidemiology and Biostatistics, 526 Norfolk Place, London W2 1PG, London, UK*
[f] *MRC-PHE Centre for Environment and Health, St. George's University of London, Population Health Research Institute, Cranmer Terrace, SW17 0RE, London, UK*

Contents

- S1. General framework
- S2. Prediction details
- S3. Time series plot of respiratory mortality and air particle metrics
- S4. Cross-validation
- References for supplementary material

*Corresponding author: monica.pirani@kcl.ac.uk

S1. General framework

In this section we recall briefly some fundamentals of the Dirichlet process mixture model.
For simplicity, we start setting out briefly a standard finite mixture model (McLachlan and Peel, 2000; Fraley and Raftery, 2002; McLachlan and Baek, 2010) which is a probabilistic approach to clustering that works by partitioning a data set according to statistical features shared by members of the same group. Let a data set of random values $x = (x_1, \dots, x_n)$ be drawn independently from some unknown distributions. In a finite mixture model, the density for x is modelled as a mixture of K component densities $f_k(x;\theta_k)$ on some unknown proportions $w_1, \dots, w_K$, that is $p(x) = \sum_{k=1}^{K} w_k f_k(x, \theta_k)$. Here, $\theta = (\theta_1, \dots, \theta_K)$ are the cluster parameters and $w = (w_1, \dots, w_K)$ are the mixture probabilities (or mixing weights) which must be positive and sum to one. $f_k(\cdot, \theta_k)$ is a distribution belonging to a parametric family, characterised by finite-dimensional θ_k.
The density of x can be written as an integral $p(x) = \int p(x|\theta)F(\theta)d\theta$, where F is a mixing distribution, $F = \sum_{k=1}^{K} w_k \delta_{\theta_k}$, with δ_θ be a Dirac distribution (atom) centered at θ. To bypass the problem of choosing K, we can assume a Bayesian nonparametric approach and let $K = \infty$ (Neal, 1992; Rasmussen, 2000). Assuming that the mixture distribution, F, follows a Dirichlet process (DP), we obtain the DP mixture models (Escobar, 1994; Escobar and West, 1995).

To briefly review the DP introduced in Ferguson (1973) (for a comprehensive introduction, refer to Ghosal 2010; Neal 2000; Teh 2010), let $(\Omega, \mathbb{A})$ be a measurable space. Suppose F_0 is a probability distribution (measure) with support in space Ω and α is a positive real number. Thus, F is distributed according to the DP with base distribution F_0 and concentration parameter α, if for any finite measurable partition $(A_1, \dots, A_K)$ of Ω, with $A \in \mathbb{A}$, a random vector $(F(A_1), \dots, F(A_K))$ is distributed as a finite-dimensional Dirichlet distribution:

$$(F(A_1), \dots, F(A_K)) \sim \mathrm{Dir}(\alpha F_0(A_1), \dots, \alpha F_0(A_K))$$

In notation, we write $F \sim DP(\alpha, F_0)$ to denote that the random probability measure F follows a DP. The α parameter (often called the mass parameter) controls the number of components of the mixture and F_0 specifies the mean of the process, $E(F) = F_0$.
Draws from a DP are almost surely discrete probability distributions, thus it can be a good candidate for the probability distribution F that has been shown to be discrete with probability one (Blackwell, 1973). This feature of the DP explains its use for dimension reduction and clustering, where x_i and x_j are member of a common cluster if $\theta_i = \theta_j$.

S2. Prediction details

The main goal in performing prediction was to obtain a posterior predictive distribution of the response under a new input scenario (s) of exposure.

The posterior predictions were carried out according to the method proposed by Liverani et al. (2015) using simple allocations where, at each sweep r of the MCMC sampler, is assigned $\hat{\mu}_s^r = \mu_k^r$. In particular, an additional latent indicator variable $\hat{g}_s^r$ was defined, corresponding to each predictive scenario. Let $z_t^* = (z_{t,1}^*, \dots, z_{t,P}^*)$ be the new profile of exposure, the posterior probabilities are computed as: $p(\hat{g}_s^r = k|z_t^*, \Theta^r, y, z_t)$. Given these probabilities, a predicted averaged cluster-specific estimate of the response is performed, for each new profile of particles at each sweep: $\hat{\mu}_s^r = \sum_{k=1}^{\infty} p(\hat{g}_s^r = k|z_t^*, \Theta^r, y_t, z_t)\mu_k^r$.

S3. Time series plot of respiratory mortality and airborne particle metrics

Fig. S3.1 shows the time series of daily mortality counts for respiratory diseases and daily concentrations of airborne particle metrics from London (UK) for the years 2002-2005. The data for both mortality and particles exhibited a pronounced seasonal pattern, for example, with mortality increasing during winter months and decreasing during summer months.

Figure S3.1: Daily mortality counts and daily airborne particle metrics in London, 2002-2005.

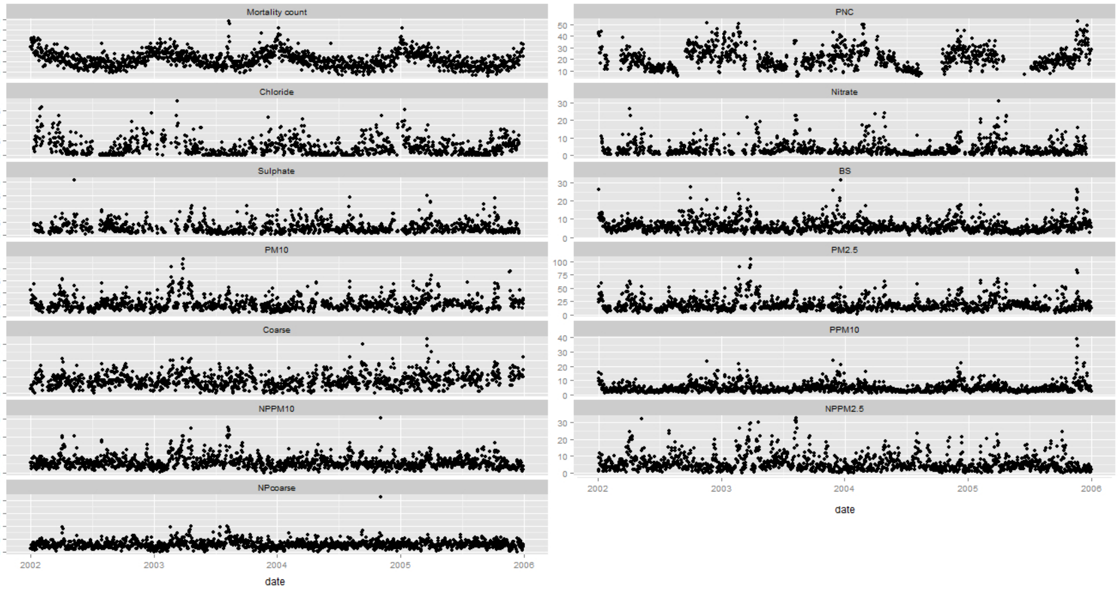

Note: Airborne particle metrics are plotted in the original measurement units as reported in the main paper.

S4. Cross-validation

We predicted the respiratory counts of deaths for the year 2005 (here used as validation sample) using the data 2002-2004 as training sample. We compared the observed mortality and validation predictions in the year 2005 using the adjusted R^2 and the root mean squared error (RMSE) given by $\sqrt{\frac{1}{T_v}\sum_{t=1}^{T_v}(y_t^* - y_t)}$, where T_v is the number of observations for the validation set (i.e., 365 days), y_t^* and y_t are respectively the predicted and observed mortality.

The cross-validation produced a R^2 of 0.61 and a RMSE of 8.92. Fig. S4.1 provides the scatter plot of validation predictions for the count number of deaths in 2005 against the corresponding observations.

Figure S4.1: Scatter plot of validation predictions against observations.

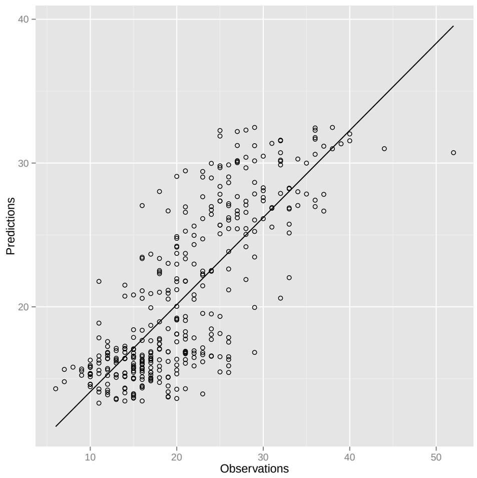

References for supplementary material

Blackwell D. Discreteness of Ferguson selections. Annals of Statistics 1973;1:356–8.

Escobar MD. Estimating normal means with a Dirichlet process prior. Journal of the American Statistical Association 1994;89:268–77.

Escobar MD, West M. Bayesian density estimation and inference using mixtures. Journal of the American Statistical Association 1995;90:577–88.

Ferguson TS. A Bayesian analysis of some non-parametric problems. The Annals of Statistics 1973;1:209–30.

Fraley C, Raftery AE. Model-based clustering, discriminant analysis, and density estimation. Journal of the American Statistical Association 2002;97:611–31.

Ghosal S. The Dirichlet process, related priors and posterior asymptotics. In: Hjort NL, Holmes C, Müller P, Walker SG, editors. Bayesian Nonparametrics. Cambridge University Press; 2010. p. 35–79.

Liverani S, Hastie DI, Azizi L, Papathomas M, Richardson S. PReMiuM: an R package for profile regression mixture models using Dirichlet processes. Journal for Statistical Software 2015; forthcoming, Available at http://arxiv.org/abs/1303.2836.

McLachlan GJ, Baek J. Clustering of high-dimensional data via finite mixture models. In: Fink A, Lausen B, Seidel W, Ultsch A, editors. Advances in Data Analysis, Data Handling and Business Intelligence. Springer-Verlag; 2010. p. 33–44.

McLachlan GJ, Peel D. Finite Mixture Models. John Wiley & Sons, Inc, 2000.

Neal RM. Bayesian mixture modeling. In: Maximum Entropy and Bayesian Methods: Proceedings of the 11th International Workshop on Maximum Entropy and Bayesian Methods of Statistical Analysis. Seattle; 1992. p. 197–211.

Neal RM. Markov chain sampling methods for Dirichlet process mixture models. Journal of Computational and Graphical Statistics 2000;9:249–65.

Rasmussen CE. The infinite Gaussian mixture model. In: Solla SA, Leen TK, Müller KR, editors. In Advances in Neural Information Processing Systems 12. MIT Press; 2000. p. 554–60.

Teh YW. Dirichlet processes. In: Encyclopedia of Machine Learning. Springer; 2010. .
